# Evaluating the burden of respiratory tract infections among mortality cases in Karachi, Pakistan: a post-pandemic surveillance analysis

**DOI:** 10.7189/jogh.15.04198

**Published:** 2025-08-04

**Authors:** Furqan Kabir, Raheel Allana, Inci Yildirim, Aneeta Hotwani, Sameer M Belgaumi, Fatima Aziz, Fauzia Aman Malik, Saima Jamal, Obianuju Aguolu, Nazia Ahsan, Zahra Hasan, Shabina Ariff, Saad B Omer, Abdul Momin Kazi

**Affiliations:** 1 Department of Paediatrics, Aga Khan University, Karachi, Pakistan; 2 Department of Pediatric Infectious Diseases, Yale School of Medicine, New Haven, USA; 3 Peter O’ Donnell Jr School of Public Health, UT Southwestern Medical Centre, Texas, USA; 4 Division of Epidemiology, Public Health Department, Ohio State University, Columbus, USA

## Abstract

**Background:**

Respiratory tract infections (RTIs) significantly impact global health, but particularly affect low- and middle-income countries. They contribute to morbidity and mortality, especially among vulnerable populations. We evaluated the burden of RTIs in mortality cases in an urban slum of Karachi, Pakistan, during the post-COVID-19 pandemic period.

**Methods:**

We conducted a prospective observational study from September 2022 to October 2023 in Ali Akbar Shah, Karachi. We collected 350 nasal swabs from deceased individuals and tested them for severe acute respiratory syndrome coronavirus 2 (SARS-CoV-2) and other respiratory pathogens using reverse transcription polymerase chain reaction (RT-PCR) and the TaqMan Array Card (TAC) assay. Additionally, we performed verbal autopsies to determine the cause of death.

**Results:**

Most deaths occurred at home (n/N = 234/350, 66.8%). Hospital-based deaths were more common among children under five years of age (n/N = 81/132, 61.3%), while individuals over five were more likely to die at home (n/N = 180/211, 85.3%). In the post-pandemic period, 6% (n/N = 21/350) of deceased individuals tested positive for COVID-19. The TAC assay analysis found *Klebsiella pneumoniae* (n/N = 150/350, 42.8%), *Staphylococcus aureus* (n/N = 141/350, 40.3%), and *Streptococcus pneumoniae* (n/N = 106/350, 30.3%) to be the most common pathogens. Co-infections were common, with 90.4% of COVID-19-positive cases also harbouring other respiratory pathogens.

**Conclusions:**

We observed a high burden of RTIs in Karachi, with *Klebsiella pneumoniae* playing a major role in overall mortality across all age groups. Co-infections with multiple respiratory pathogens were common, underscoring the need for better diagnostic and treatment strategies. Improved surveillance and potential vaccine development for *Klebsiella pneumoniae* and other notable pathogens could reduce mortality in similar settings. However, limitations such as post-mortem colonisation, contamination, and the absence of histopathologic confirmation necessitate cautious interpretation of pathogen-related mortality.

Respiratory tract infections (RTIs) encompass a wide range of upper and lower respiratory tracts illnesses caused by viruses, bacteria, or other pathogens [1]. They are a significant global public health concern due to their widespread prevalence, severe complications, and impact on vulnerable populations such as the elderly, young children, immunocompromised individuals, and those with underlying health conditions [2,3]. Lower RTIs impose the highest burden on human health of all RTIs, incurring twice as many disability-adjusted life years compared to ischaemic heart disease and diabetes mellitus [4].

Pathogens, meanwhile, not only cause primary respiratory infections such as pneumonia, but they also play a role in the exacerbation of chronic lung diseases, including chronic obstructive pulmonary disease, interstitial lung disease, and bronchiectasis. Importantly, in low- and middle-income countries, lower RTIs are still the most common cause of mortality in children under five years of age [5,6]. This has been linked to poverty, which is associated with malnutrition, as well as overcrowding and air pollution, which increase the risk of infection with airborne pathogens [7].

The COVID-19 pandemic further highlighted the critical importance of respiratory infection surveillance and control [8,9]. Understanding the epidemiology and burden of RTIs in its aftermath is now important for several reasons [9]. First, given the increased diagnostic challenges stemming from the co-circulation of other respiratory viruses and the ensuing strain on healthcare, there is a need to comprehensively understand the impact of RTIs on healthcare systems [10]. Second, the influence of the COVID-19 pandemic on RTI cases, clinical outcomes, and healthcare resource use needs to be evaluated to inform future pandemic preparedness strategies and resource allocation [10]. Finally, the post-pandemic period presents an opportunity to assess changes in healthcare-seeking behaviours, vaccination coverage, and healthcare infrastructure that may have long-lasting implications for RTI detection and management.

It is crucial to understand how these pathogens contribute to mortality post-pandemic, especially in resource-constrained urban settings. Further, limited data exist on the aetiological burden of respiratory pathogens in deceased individuals outside of hospital settings. Therefore, we sought to address the following research question: ‛What is the burden of RTIs and the distribution of specific respiratory pathogens among deceased individuals in an urban slum of Karachi, Pakistan, in the post-COVID-19 period?’ We hypothesised that RTIs, particularly those caused by bacterial pathogens, remain a major contributor to mortality in post-pandemic urban slum settings, with frequent co-infections complicating disease outcomes. Our objective was to estimate the prevalence of severe acute respiratory syndrome coronavirus 2 (SARS-CoV-2) and other respiratory pathogens among deceased individuals in a post-pandemic context; to identify the most common respiratory pathogens contributing to mortality in an urban slum population; to assess the frequency and nature of coinfections among COVID-19-positive deceased individuals; and to generate evidence for improved diagnostic, treatment, and prevention strategies, including the potential role for vaccines against high-burden bacterial pathogens.

## METHODS

### Study design and setting

We conducted a prospective, observational surveillance study in Ali Akbar Shah, an urban slum in Karachi, Pakistan, with a population of approximately 105 300 [11]. The area is covered by the Aga Khan University's Health and Demographic Surveillance System, which includes one primary healthcare centre [11] and comprises 195 blocks, each consisting of 200 to 250 structures (*i.e.* buildings with a single entrance and a boundary, with each having a unique identifier). Surveillance is conducted on married women of reproductive age, with community healthcare workers (CHWs) updating the information every two months.

This study is a part of the broader ‛COVID-19, Burial Site Surveillance to Measure Excess Mortality – Pakistan’ project funded by the Bill & Melinda Gates Foundation. In preparation for the aforementioned project, we established relationships with the community leaders and religious leaders in the target population to facilitate the process of obtaining information regarding death. The study lasted 13 months, from September 2022 to October 2023.

### Study participants, eligibility, and recruitment

Study participants were recently deceased individuals from within the Health and Demographic Surveillance System catchment area for whom the death notification, family consent, and sample collection occurred within 24 hours of death. The sample collection timeline required by the laboratory aligns with the cultural and religious norms of the community.

We obtained informed consent through a culturally sensitive, community-engaged approach to ensure ethical transparency in this vulnerable population. First, as a part of sustained engagement efforts, we approached community members with the help of trusted local intermediaries such as CHWs and religious leaders who helped introduce the study to community members. Our research team then explained the purpose of the study, the procedures for post-mortem nasal swab collection, potential implications, and the voluntary nature of participation in their native language using a standardised script. For illiterate participants, we employed a witnessed verbal consent process to ensure comprehension. Feedback from focus group discussions conducted by study team indicated initial concerns about post-mortem sampling, which were mitigated through continued dialogue, respect for cultural and religious practices, and timely, dignified sample collection conducted by study team before funeral rituals commenced. This approach encouraged trust and facilitated acceptance of the procedure across the study population. The study clinical staff performed nasopharyngeal swab collection within a short window after death to preserve sample quality, but only after obtaining explicit consent. Following Islamic practices, the body was treated respectfully, with procedures designed not to interfere with funeral preparations.

We identified potential study participants using death reports from multiple community sources, including local CHWs, council members, religious leaders, and family members. We aimed to collect approximately 30–50 nasal swab samples per month from deceased community members of consenting households, following death alerts from key informants, until reaching a total of 350 samples over the study duration. We targeted to collect 350 samples based on feasibility considerations informed by prior mortality surveillance in the same setting, as well as operational capacity for specimen collection and testing. Although we did not conduct a formal power calculation due to the exploratory nature of this surveillance study, we deemed the sample size to be sufficient for detecting meaningful patterns in pathogen prevalence and co-infection trends, including the detection of less common respiratory pathogens. We tested all samples for SARS-CoV-2 using reverse transcription polymerase chain reaction (RT-PCR) and for common respiratory pathogens using customised TrueMark Respiratory Panel 2.0, TaqMan Array Card (TAC) assay (ThermoFisher Scientific, US). Additionally, we performed genomic sequencing of SARS CoV2 on 75 of these samples.

### Religious and contextual considerations

In the Muslim community, adherence to specific burial practices is crucial. Before burial, a body is ritually washed, involving the use of cotton to clean the nostrils of the deceased. Our sampling approach aligns with these practices, as indicated by data from in-depth interviews with key informants [12]. We obtained approval from religious scholars, affirming the religious acceptability of collecting nasopharyngeal swabs during the ritual bath. Additionally, we acquired a *fatwa* (an approval or religious ruling from religious scholars) to affirm that collecting specimens via nasopharyngeal swabs after death is religiously acceptable in the context of the Islamic, Christian, and Hindu faiths. We conveyed death alerts to the team from key community stakeholders, such as religious leaders, traditional birthing attendants, and graveyard personnel.

### Sample collection and storage

Upon receiving a death notification, the study team travelled to the identified household, obtained informed verbal consent, completed a case record form, and performed nasal swab collection from the deceased. For nasopharyngeal swabs, a flexible wire shaft mini-tip swab was inserted through the nares parallel to the palate until resistance was felt, ensuring contact with the nasopharynx. The swab was gently rubbed and rolled, left in place for several seconds to absorb secretions, and then removed while rotating it. For nasal swabs, a single polyester swab with a plastic shaft was used to sample both anterior nares. The swab was inserted into the nostril, rotated, left in place for five seconds to absorb secretions, and then slowly withdrawn. We collected samples from each participant using both dry nasopharyngeal swabs and standard nasopharyngeal swabs stored in viral transfer medium (wet swabs). A team member conducted the procedure wearing full healthcare-grade personal protective equipment (PPE) to minimise exposure. To ensure sample integrity, swabs were transported at 2–8°C to an infectious diseases research laboratory at Aga Khan University, where they were tested for SARS-CoV-2 using RT-PCR and analysed through amplicon sequencing for variant detection. Samples from both standard and dry nasal swabs were stored at –80°C. We processed dry swabs by adding 2.5 mL PBS, vortexed, incubated, and then extracted RNA using the QIAamp Viral RNA Mini Kit. We tested RNA using the FDA-approved COBAS® SARS-CoV-2 RT-PCR assay; VTM samples were also eligible for clinical testing. These protocols were adapted from World Health Organization (WHO) guidelines for post-mortem respiratory sampling in low-resource settings to minimise degradation and contamination. We tested samples for SARS-CoV-2 RNA and other viral respiratory pathogens *via* RT-PCR and TAC assay, respectively.

### Verbal autopsy procedure

Following verbal consent for verbal autopsy, we interviewed families of the deceased at least one week after burial, to ensure timely and accurate recollection of symptoms and events leading to death using the updated WHO verbal autopsy questionnaire 2016, including COVID-19 questions. Two trained physicians reviewed each completed verbal autopsy and assigned a probable cause of death based on symptomatology and history. In cases where discrepancies arose between the two reviewers, a third senior physician reviewed the case and provided the final adjudication. We implemented this approach to enhance reliability and transparency in the classification of the causes of death. In addition, a trained psychologist accompanied the physicians during verbal autopsy visits, provided grief support to parents, caregivers, or relatives of the deceased during the same visit as the verbal autopsy form administration and conducted regular counselling sessions with the study team as well to help them cope with the emotional toll of frequent interactions with bereaved families [13].

### Laboratory evaluations

Samples collected from both standard and dry nasal swabs were stored at −20°C until processing. We conducted elution and nucleic acid extraction from the swabs inside a biosafety cabinet in a BSL-3 laboratory. For the standard dry swabs, material elution involved adding 2.5 mL of PBS to a 4.5 mL Nunc Cryovial tube, vortexing for 30 seconds, and incubating for 10 minutes before viral RNA extraction and SARS-CoV-2 PCR [14]. Alternatively, we sent eluted samples for PCR in the clinical laboratory. Spun polyester swab nasal samples placed in VTM tubes were assessed in the clinical laboratory for SARS-CoV-2 using RT-PCR, and these swabs in VTM tubes were also eligible for routine clinical laboratory testing. For SARS-CoV-2 sequencing, the ARTIC-NEB: SARS-CoV-2 Library Prep V.4 protocol was followed, involving obtaining amplicons from extracted RNA, creating an amplicon library with the FS DNA Library Prep Kit, and pair-end sequencing on the Illumina iSEQ100 platform, with the IDseq pipeline generating the consensus genome [15]. Additionally, nasopharyngeal swabs underwent SARS-CoV-2 RT-PCR in the clinical laboratory using the FDA-approved COBAS® SARS-CoV-2 RT-PCR assay, with sequencing following the ARTIC-NEB protocol [16]. For multiplex testing, the TaqMan Array Card (TAC) assay was used, which included 44 TAC assays optimised for 41 respiratory tract microbes, including SARS-CoV-2 [17]. Total nucleic acid (TNA) extraction was performed using the Roche Magna Pure extractor, and the PCR reaction mix was prepared using TNA, TaqPath™ 1-Step RT-qPCR Master Mix (ThermoFisher Scientific, US) and TaqMan™ Fast Advanced Master Mix, (ThermoFisher Scientific, US) [18]. The TAC assay was run on the Applied Biosystem Quant Studio 7 system based on the real-time PCR principle as detailed in Liu and Kabir et al.'s study [19].

### Data management and analysis

We securely managed the data collected during the study to ensure confidentiality and integrity. We anonymised all participant information, assigning unique identifiers to each case. We performed data entry using a secure, web-based in-house application for managing research data. Only authorised personnel could access the database through password-protected accounts, and routine backups were conducted to prevent data loss.

We calculated descriptive statistics for demographic characteristics, clinical symptoms, and pathogen distribution. Categorical variables (*e.g.* gender, location of death, presence of coinfection) were summarised using frequencies and percentages. We used χ^2^ or Fisher exact test (for small cell counts) to compare categorical variables across age groups (*e.g.* place of death, presence and severity of antemortem respiratory symptoms), whereby a *P*-value of <0.05 indicated statistical significance. Further, we defined co-infection as the detection of two or more respiratory pathogens (bacterial or viral) from the same individual’s post-mortem sample. Importantly, detection by PCR indicates the presence of nucleic acid material but does not distinguish between active infection, colonization, or contamination. We calculated the co-infection rate by dividing the number of cases with co-infections by the total number of cases and multiplying by 100, using the formula:

Co-infection rate = (cases with co-infections/total number of cases) × 100

Total cases = (17/21) × 100.

We analysed RT-PCR results for respiratory pathogens to identify prevalence and distribution trends, and further stratified the causes of death from verbal autopsy coding to explore mortality patterns.

We performed our statistical analysis in Stata, version 16 (StataCorp LLC, College Station, TX, USA).

## RESULTS

**Figure 1:**
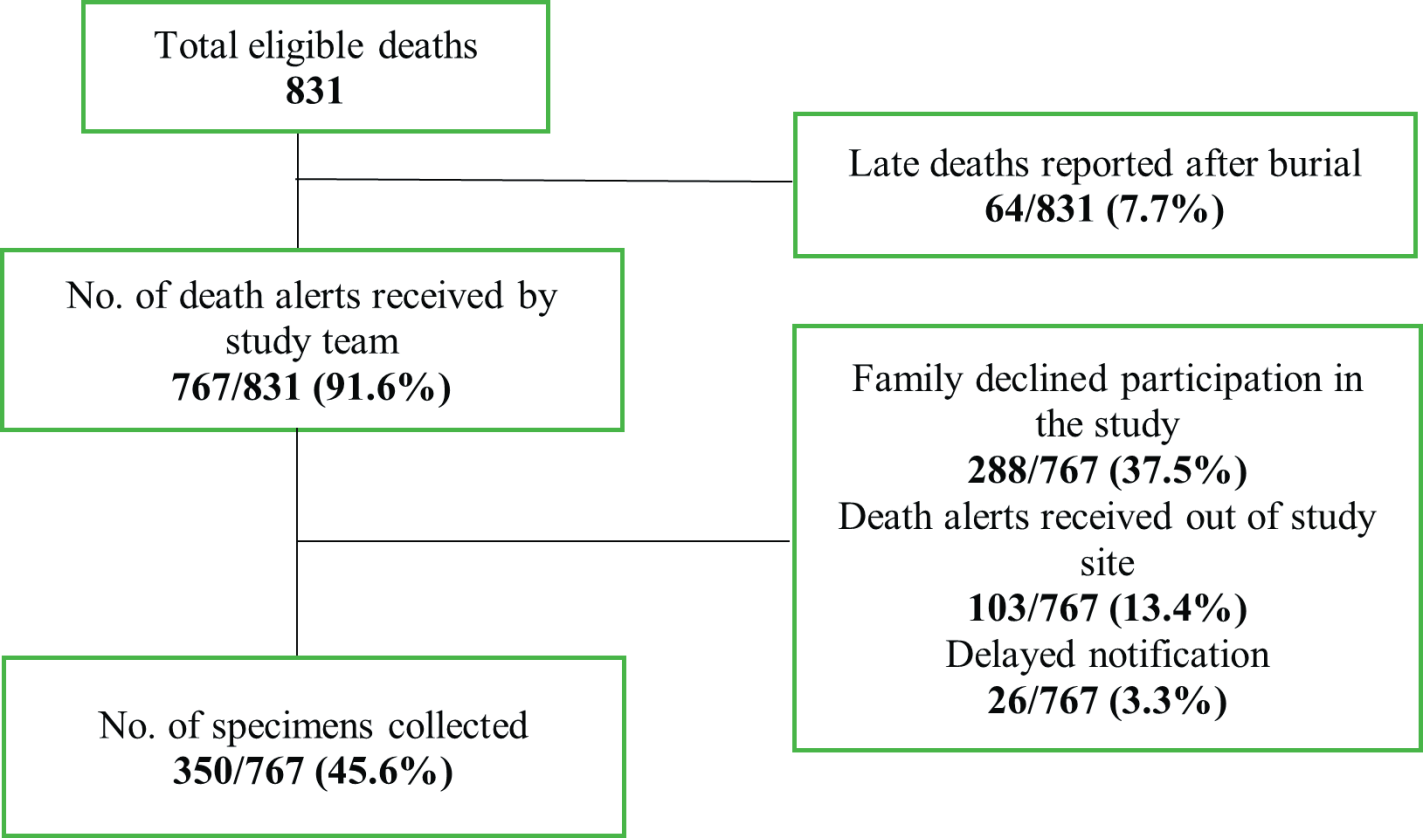
Sampling strategy of the nasal swab collection process.

Out of 831 eligible deaths, 767 (91.6%) were reported to the study team, but 64 of 831 deaths (7.7%) were reported late after the burial. Among the reported cases, 288 (37.5%) declined participation, 103 (13.4%) were outside the study site, and 26 (3.3%) were delayed notifications. Nasal swabs were successfully collected from 350 cases, representing 45.6% of reported deaths. Out of 350 samples, n/N = 21/350 (6%) tested positive for COVID-19 (**Figure 1**). Most of the cases occurred among men (n/N = 215/350, 61.4%). More than half of the deaths occurred at home (n/N = 234/350, 61.1%), with a significant difference across age groups (<5, 5–18, >18 years; *P*-value = 0.001). Family members reported respiratory symptoms in nearly one quarter of all deaths, and of those, two-thirds were reported as very severe (**Figure 1**, **Table 1**).

**Table 1:** Demographic characteristics of deceased individuals in Karachi Pakistan, September 2022 to October 2023*

	Age at death				
	**<5 years (n = 132, 37.7%)**	**5–18 years (n = 7, 2.0%)**	**>18 years (n = 211, 60.2%)**	**Total**	***P*-value**
**Sex**					0.42
Male	78 (59.0)	3 (42.8)	134 (63.5)	215 (61.4)	
Female	54 (40.9)	4 (57.1)	77 (36.4)	135 (38.5)	
**Place of death**					0.001
Home	51 (38.6)	3 (42.8)	180 (85.3)	234 (66.8)	
Hospital	81 (61.3)	4 (57.1)	31 (14.6)	116 (33.1)	
**Antemortem respiratory symptoms**					0.001
No	99 (75.0)	4 (57.1)	139 (65.8)	242 (69.1)	
Yes	33 (25.0)	3 (42.8)	72 (34.1)	77 (22.0)	
Mild to moderate	28 (21.2)	3 (42.8)	46 (21.8)	77 (10.4)	
Severe	15 (11.3)		4 (1.8)	19 (24.7)	
Very severe	12 (9.0)	1 (14.2)	37 (17.5)	50 (64.9)	
Not documented	99 (75.0)	4 (57.1)	139 (65.8)	242 (69.1)	

*Presented as n (%).

The TAC assay found *Klebsiella pneumoniae* (n/N = 150/350, 42.8%) and *Staphylococcus aureus* (n/N = 141/350, 40.3%) to be the most common microorganisms, followed by *Streptococcus pneumoniae* (n/N = 106/350, 30.2%) and *Hemophilus influenzae type B* (n/N = 101/350, 28.8%) (Table S1 in the **Online Supplementary Document**).

We analysed the distribution of respiratory pathogens by age group and place of death. Among children under five years (n = 132, 37.7%), most deaths occurred in hospitals (n = 81, 61.3%), while the remainder occurred at home (n = 51, 38.7%). *Klebsiella pneumoniae* was the most prevalent pathogen, identified in 28 (21.2%) home deaths and 45 (34.9%) hospital deaths. *Staphylococcus aureus* was found in 17 (12.8%) home deaths and 30 (22.7%) hospital deaths. Among individuals aged 5–18 years (n = 7, 2.0%), hospital deaths (n = 4, 57.2%) were slightly more frequent than home deaths (n = 3, 42.8%). *Klebsiella pneumoniae* was detected in 2 (28.5%) home deaths and 3 (42.8%) hospital deaths, while *Staphylococcus aureus* was present in both settings (n = 3, 42.8%). Of adults over 18 years (n = 211, 60.2%), the majority died at home (n = 180, 85.3%) rather than in hospitals (n = 31, 14.7%). *Klebsiella pneumoniae* was detected in 52 (24.6%) home deaths and 20 (9.5%) hospital deaths, while *Staphylococcus aureus* was identified in 59 (27.9%) home deaths and 29 (13.7%) hospital deaths (**Table 2**).

**Table 2:** Distribution of respiratory pathogens by age and place of death

Age group	<5 years (n = 132, 37.7%)		5–18 years (n = 7, 2.0%)		>18 years (n = 211, 60.2%)	
**Place of death**	**Home (51, 38.6%)**	**Hospital (81, 61.3%)**	**Home (3, 42.8%)**	**Hospital, (4, 57.1%)**	**Home (180, 85.3%)**	**Hospital (31, 14.7%)**
*Klebsiella pneumoniae*	28 (21.2)	45 (34.9)	2 (28.5)	3 (42.8)	52 (24.6)	20 (9.5)
*Staph aureus*	17 (12.8)	30 (22.7)	3 (42.8)	3 (42.8)	59 (27.9)	29 (13.7)
*Hemophilus influenzae type B*	18 (13.6)	25 (18.9)	0 (0.0)	2 (28.5)	37 (17.5)	19 (9.0)
*Moraxella catarrhalis*	17 (12.8)	16 (12.1)	0 (0.0)	1 (14.2)	40 (18.9)	10 (4.7)
*Streptococcus pneumoniae*	17 (12.8)	16 (12.1)	0 (0.0)	1 (14.2)	58 (27.4)	14 (6.6)

*Presented as n (%).

Among viruses, rhinovirus 1 had the highest frequency with 73.0% in children <5 years and 75.0% in those aged 5–18 years. Furthermore, human herpesvirus 6 and human herpesvirus 5 were mostly observed in hospital deaths, with 82.3% in adults >18 years.

The leading causes of death in children under five were perinatal asphyxia (n/N = 21/132, 15.9%), neonatal sepsis (n/N = 18/132, 13.6%) and preterm birth complications (n/N = 11/132, 8.3%). In 5–18-year-olds, acute respiratory infection (n/N = 2/7, 28.6%) was the primary cause of death, while in adults >18 years, acute cardiac disease (n/N = 54/211, 25.5%), liver cirrhosis (n/N = 26/211, 12.3%), and stroke (n/N = 21/211, 9.9%) were prominent. Other notable causes of death in adults included diarrhoeal disease (n/N = 14/21, 6.6%) and chronic obstructive pulmonary disease (n/N = 7/211, 3.3%) (Table S2 in the **Online Supplementary Document**).

Among the 21 COVID-19 positive cases, 17 cases had at least one co-infection with viral or bacterial pathogens. Out of the 21 individuals who tested positive for COVID-19 via post-mortem PCR testing, 3 (14.3%) reported acute cardiac disease as the cause of death determined through verbal autopsy.

Based on this calculation, the co-infection rate was 80.95%, indicating a high frequency of multiple infections among the cases (**Table 3**).

**Table 3:** Co-infection in PCR-positive deceased participants (n = 21)

					Co-infections												
**Age at death in years**	**Cause of death**	**Place of death**	**Epstein-Barr virus**	**Cytomegalovirus**		**Human herpesvirus 6**	**RSV virus B**	**Enterovirus D68**	**Rhinovirus 1**	**Rhinovirus 2**	**SARS-COV-2S**	**SARS-COV-2N**	***Haemophilus influenzae***	***Klebsiella pneumoniae***	***Moraxella catarrhalis***	***Staphylococcus aureus***	***Streptococcus pneumoniae***
73	COPD	Hospital														*	
10	Accidental/drowning	Home											*			*	
45	Acute cardiac disease	Hospital												*	*	*	*
62	Acute cardiac disease	Home												*			
70	Acute cardiac disease	Hospital	*													*	
<1	Congenital heart disease	Hospital		*									*	*	*		*
65	Diabetes mellitus	Hospital															
80	Diabetes mellitus	Home		*										*			
99	Diarrhoeal disease	Home									*				*	*	*
45	Drug addiction	Home												*			*
31	Drug addiction	Home												*			
65	Intestinal cancer	Hospital									*			*			
45	Liver cancer	Home									*	*		*		*	
<1	Measles	Hospital					*		*	*				*			
<1	Neonatal sepsis	Hospital												*			
62	Not specified	Hospital												*		*	
72	Not specified	Home											*	*	*		
100	Not specified	Home															
40	Oral neoplasm	Home				*					*	*		*		*	
80	Stroke	Hospital								*						*	
75	Stroke	Home						*							*	*	*

COPD – chronic obstructive pulmonary disease, RSV – respiratory syncytial virus

## DISCUSSION

We found distinct patterns in respiratory pathogen distribution and cause of death across age groups and by place of death. Among respiratory pathogens, *Klebsiella pneumoniae* was the most prevalent (42.8% of cases), followed by *Staphylococcus aureus* (40.2%). *Klebsiella pneumoniae* was slightly more prevalent in hospital deaths compared to home deaths. In contrast, *Streptococcus pneumoniae* and *Moraxella catarrhalis* were more commonly detected in deaths at home. *Moraxella catarrhalis* was notably identified in home deaths among adults, especially in two-thirds of cases aged over 18 years. Age-stratified analysis showed distinct aetiologies, with children under five primarily succumbing to perinatal asphyxia (15.9%), neonatal sepsis (13.6%), and preterm complications (8.3%). The 5–18 years group showed a predominance of acute respiratory infections (28.5%) and pulmonary tuberculosis (14.2%), while adults >18 years were more affected by acute cardiac disease (25.5%), liver cirrhosis (12.3%), and stroke (9.9%). The greater proportion of deaths at home may reflect significant barriers to accessing healthcare services, potentially leading to delays in diagnosis and treatment, especially in urban slum settings where healthcare access is often limited.

The TAC assay results for pathogen composition in DNS samples provided insights into the diversity of various microbial species. In our study, the most common pathogens identified in mortality cases were *Klebsiella pneumoniae, Staphylococcus aureus, Streptococcus pneumoniae*, and *Haemophilus influenzae type B.* Our results are consistent with the estimates of the global burden of RTIs by the Global Burden of Disease 2019 Antimicrobial Resistance Collaborators [20], in which *Streptococcus pneumoniae, Staphylococcus aureus*, and *Klebsiella pneumoniae* were the major contributors to mortality for all ages, while co-infections, particularly with *Klebsiella pneumoniae,* were common (42.8%). Our results are also consistent with Sreenath and colleagues' study conducted in India [21], where *Klebsiella pneumoniae* was found to be a major pathogen co-infected with other pathogens, *i.e.* 41.4% (n/N = 79/191). However, while we focussed on mortality samples, Sreenath and colleagues' study examined COVID-19-admitted patients during the global pandemic.

We found *Klebsiella pneumoniae* in 51% of nasopharyngeal samples for the deceased under five years of age. These results are consistent with Child Health and Mortality Prevention Surveillance data in sub-Saharan Africa and South Asia, where over half of all the deaths were caused by *Klebsiella pneumoniae* detected by TAC [22]. The epidemiology of *Klebsiella pneumonia* as a nosocomial pathogen is well characterised, and nosocomial outbreaks in neonatal intensive care contribute importantly to hospital-acquired infections in children [23]. However, we also observed deaths with *Klebsiella pneumonia* that occurred in the community among children with limited or no contact with healthcare facilities. Data on community-acquired *Klebsiella pneumonia* infections in children is limited, and our findings demonstrate the need for further research in this area. Additionally, distinguishing the exact role of *Klebsiella pneumonia* in a child’s death based on post-mortem nasal swab specimens is difficult because it commonly colonises the gastrointestinal tract and nasopharynx, so detection of the pathogen post-mortem could potentially reflect agonal spread, post-mortem translocation, post-mortem overgrowth, or contamination [24].

There was an 80% co-infection rate in our sample of 350 swabs. These findings are consistent with a study by Swamy and colleagues in India, in which the overall positivity for viral and bacterial pathogens was found to be 76.7% and another study by Bhuyan and colleagues from Bangladesh, where the positivity rate was 82.5% [25]. Furthermore, the co-infection rate among COVID-19 deceased cases (n = 21) was 90.4%. This is consistent with a study in China, where 94.2% of COVID-19 patients had co-infections [26]. Zhang and colleagues also reported bacterial coinfections in 7.7% of patients with SARS-CoV-2 [27], while a French study reported 28% coinfections at ICU admission of patients with COVID-19 [28]. Moreover, a meta-analysis has highlighted a somewhat high rate (23.5%) of fungal-bacterial co-infections and super-infections in COVID-19 patients [29]. Among 21 deceased individuals who tested positive for COVID-19 via post-mortem PCR, three cases (14.3%) were determined based on verbal autopsy data to have died due to acute cardiac disease. This aligns with previous studies reporting that preexisting cardiovascular disease and evidence of cardiac injury are associated with higher mortality in hospitalised COVID-19 patients [30,31]. Similar to our study, Page and colleagues have shown that SARS-CoV-2 contributes to cardiac complications through various mechanisms, including systemic inflammation, endothelial dysfunction, prothrombotic states, and possible direct myocardial involvement [32]. The above-mentioned studies have focussed on live individuals, whereas our study uniquely evaluated only mortality cases, with clinical findings assessed post-mortem. Our findings also suggest a potential link between COVID-19 infection and acute cardiac events. This allowed us to examine factors present after death, such as nosocomial infections and the timing of sample collection. By including both community (home) and hospital deaths, we were able to capture a broader spectrum of disease severity, infection patterns, and contributing factors. Such differences may also reflect varying case definitions, diagnostic platforms (*e.g.* PCR *vs.* culture), timing of sample collection, and age group distributions. Additionally, disparities in healthcare access, underlying comorbidities, and local epidemiological trends likely influenced pathogen prevalence, which must be considered when comparing our findings with other studies.

Our study indicated that among viruses, rhinovirus 1 was the most predominant virus with a frequency of 20%. Our results are consistent with the study by Lin and colleagues, where virus co-infection mainly includes respiratory viruses such as enterovirus/rhinovirus (hRV) [33]. While hRV is commonly considered a coloniser, particularly in adults, emerging evidence suggests that its presence may have clinical significance, especially among vulnerable populations. Recent studies have highlighted the potential pathogenic role of hRV in adults, particularly the elderly and those with underlying health conditions. For instance, Fica and colleagues found that hRV infections in elderly patients were associated with higher morbidity and mortality compared to influenza virus infections, including increased rates of pneumonia and longer hospital stays [34]. Moreover, hRV infections have been shown to alter respiratory microbiota, potentially facilitating secondary bacterial infections. Studies have observed that hRV infection can lead to increased colonisation by bacteria such as *Streptococcus pneumoniae*, *Haemophilus influenzae*, and *Moraxella catarrhalis*, which are known to cause severe respiratory infections [35]. These findings suggest that, in certain contexts, hRV detection in post-mortem swabs may reflect a clinically significant infection rather than mere colonisation. Therefore, we believe that the presence of hRV warrants further discussion regarding its potential role in the pathogenesis of fatal respiratory illnesses, particularly among vulnerable populations. One notable finding was the 6.0% positive rate for COVID-19 among deceased individuals during the study period. This rate, coupled with the higher proportion of positive cases among males and individuals aged 60 and above, underscores the vulnerability of certain demographic groups to severe outcomes. The observation of a significant number of deaths occurring at home also highlights potential challenges in accessing healthcare facilities, emphasising the need for targeted interventions and community outreach programs.

### Strengths and limitations

One of the key strengths of this study is its focus on deceased individuals – a population that has not been extensively studied in the region. This has provided relevant insights into post-mortem pathogen composition and mortality patterns, offering important epidemiological data that can inform public health interventions. However, the study was conducted in a specific urban slum population in Karachi, Pakistan, which may limit the generalisability of the findings to other populations with different demographic characteristics. Such extrapolation should be done with caution while considering potential differences in healthcare access, burial practices, and community dynamics. Furthermore, although we made efforts to align the sampling method with religious burial practices, variations in religious beliefs and cultural norms within the Muslim community may exist, impacting the acceptability and feasibility of nasopharyngeal swab collection during ritual bathing. These differences could affect the applicability of the study to other Muslim communities with varying cultural contexts.

We also acknowledge several methodological limitations inherent to our study. First, relying on post-mortem nasopharyngeal swabs poses challenges in distinguishing between mere colonisation and true infection, as post-mortem microbial translocation and contamination can affect results. Therefore, attributing causality based solely on these findings requires caution. Integrating clinical history, histopathological data, and comprehensive microbiological analyses is essential for more accurate interpretations [34]. Second, our dependence on community death notifications may have introduce selection bias. Cultural norms and stigma can lead to underreporting of deaths, particularly those occurring outside formal healthcare settings. Logistical challenges, such as limited mobile connectivity and the necessity to conduct verbal autopsies within culturally acceptable time frames, may have further affected data completeness and timeliness. These factors could have led to our underestimation of certain causes of death. Lastly, low consent rates and delayed death notifications might have introduced systematic errors, potentially leading to underrepresentation of specific demographics or causes of death. We recognise these constraints as inherent to conducting research in underserved, resource-constrained peri-urban settings, and have accounted for them in our interpretation of the findings.

## CONCLUSIONS

Our findings provide insights into respiratory and COVID-19-related outcomes among deceased individuals in an urban slum population in Karachi, Pakistan. They highlight the vulnerability of certain demographic groups, fluctuations in disease prevalence over time, and the presence of co-infections, particularly with *Klebsiella pneumoniae*. Further research is needed to better understand the source of infection and risk factors for *Klebsiella pneumoniae* mortality. Given the burden of this pathogenin both children and adults, improved strategies are needed to rapidly identify those infected. Advanced genomic testing, such as metagenomic next-generation sequencing, is recommended to identify any potential new strains or pathogens that may be overlooked by current diagnostic method, such as the ones we used in this study. Our data also suggest a potential impact of developing and using effective *Klebsiella pneumoniae* vaccines in reducing child and adult mortality.

## Additional material


Online Supplementary Document
