# Supplementary material for: Evaluating the burden of respiratory tract infections among mortality cases in Karachi, Pakistan: a post-pandemic surveillance analysis
Source: J Glob Health. 2025 Aug 4;15:04198. doi: 10.7189/jogh.15.04198 (PMC12319414; doi:10.7189/jogh.15.04198)
Supplement: Online Supplementary Document [file jogh-15-04198-s001.pdf]

Supplement to: Kabir F, Allana R, Yildirim I, Hotwani A, Belgaumi SM, Aziz F, Malik FA, Jamal S, Aguolu O, Ahsan N, Hasan Z, Ariff S, Omer SB, Kazi AM. Evaluating the burden of respiratory tract infections among mortality cases in Karachi, Pakistan: a post pandemic surveillance analysis. J Glob Health. 2025;15:04198.

Table S1. Frequency and Percentage Distribution of Bacterial, Viral and Fungal Pathogens Identified in Mortality Cases

Pathogens	Frequency	%
**Bacteria**		
Klebsiella pneumoniae complex	150	42.8
Staphylococcus aureus	141	40.2
Streptococcus pneumoniae	106	30.2
Hemophilus influenzae	101	28.8
Moraxella catarrhalis	84	24.0
Chlamydophila pneumoniae	3	0.8
Bordetella pertussis	1	0.2
**Viruses**		
Rhinovirus 1	70	20.0
Epstein bar virus	36	10.2
Cytomegalovirus	31	8.8
Rhinovirus 2	31	8.8
Human herpesvirus 6	30	8.5
Measles	15	4.2
Human parainfluenza viruses 3	8	2.2
RSV virus B	8	2.2
Human bocavirus	6	1.7
Enterovirus D68	6	1.7
Human metapneumovirus	5	1.4
Adenovirus 2	4	1.1
Influenza A	4	1.1
Influenza A H1-2009	4	1.1
SARS COV 2S	4	1.1
Human parainfluenza viruses 1	3	0.8
SARS COV 2N	3	0.8
Adenovirus 1	2	0.5
Coronavirus NL63	2	0.5
Coronavirus OC43	2	0.5
Enterovirus	2	0.5
Varicella zoster virus	1	0.2
Human parainfluenza viruses 4	1	0.2
RSV virus A	1	0.2
Coronavirus 229E	1	0.2
Bordetella virus	1	0.2
**Fungi**		
Pneumocystis jirovecii	8	2.2

Table S2. Distribution of Causes of Death by Age Group (N = 350)

Causes of Death	Age Group<br>< 5 years (n=132)	5-18 years (n=7)	> 18 years (N=211)
Perinatal asphyxia	21 (15.9)	---	---
Neonatal sepsis	18 (13.6)	---	---
Preterm birth complications	11 (8.3)	---	---
Sepsis	10 (7.5)	---	8 (3.8)
Neonatal pneumonia	10 (7.5)	---	---
Intrapartum	7 (5.3)	---	---
Prematurity	7 (5.3)	---	---
Meningitis	6 (4.5)	---	---
Measles	6 (4.5)	---	---
Other obstetric complications	5 (3.7)	---	---
Acute respiratory Infection	---	2 (28.5)	7 (3.3)
Pulmonary Tuberculosis	---	1 (14.2)	---
Acute cardiac disease	---	1 (14.2)	54 (25.5)
Other and unspecified neoplasms	---	1 (14.2)	---
Accidental drowning and submersion	---	1 (14.2)	---
Diabetes mellitus	---	1 (14.2)	9 (4.2)
Road traffic accident	---	1 (14.2)	---
Diarrheal disease	---	1 (14.2)	14 (6.6)
Liver cirrhosis	---	---	26 (12.3)
Stroke	---	---	21 (9.9)
Renal failure	---	---	13 (6.1)
Oral neoplasm	---	---	7 (3.3)
Chronic Obstructive Pulmonary Disease	---	---	7 (3.3)
